# Targeted radionuclide therapy for head and neck squamous cell carcinoma: a review

**DOI:** 10.3389/fonc.2024.1445191

**Published:** 2024-08-22

**Authors:** Alexis M. Sanwick, Ivis F. Chaple

**Affiliations:** Department of Nuclear Engineering, University of Tennessee, Knoxville, TN, United States

**Keywords:** targeted radionuclide therapy, head and neck squamous cell carcinoma, epidermal growth factor receptor, theranostics, head and neck cancer, radioimmunotherapy

## Abstract

Head and neck squamous cell carcinoma (HNSCC) is a type of head and neck cancer that is aggressive, difficult to treat, and often associated with poor prognosis. HNSCC is the sixth most common cancer worldwide, highlighting the need to develop novel treatments for this disease. The current standard of care for HNSCC usually involves a combination of surgical resection, radiation therapy, and chemotherapy. Chemotherapy is notorious for its detrimental side effects including nausea, fatigue, hair loss, and more. Radiation therapy can be a challenge due to the anatomy of the head and neck area and presence of normal tissues. In addition to the drawbacks of chemotherapy and radiation therapy, high morbidity and mortality rates for HNSCC highlight the urgent need for alternative treatment options. Immunotherapy has recently emerged as a possible treatment option for cancers including HNSCC, in which monoclonal antibodies are used to help the immune system fight disease. Combining monoclonal antibodies approved by the US Food and Drug Administration, such as cetuximab and pembrolizumab, with radiotherapy or platinum-based chemotherapy for patients with locally advanced, recurrent, or metastatic HNSCC is an accepted first-line therapy. Targeted radionuclide therapy can potentially be used in conjunction with the first-line therapy, or as an additional treatment option, to improve patient outcomes and quality of life. Epidermal growth factor receptor is a known molecular target for HNSCC; however, other targets such as human epidermal growth factor receptor 2, human epidermal growth factor receptor 3, programmed cell death protein 1, and programmed death-ligand 1 are emerging molecular targets for the diagnosis and treatment of HNSCC. To develop successful radiopharmaceuticals, it is imperative to first understand the molecular biology of the disease of interest. For cancer, this understanding often means detection and characterization of molecular targets, such as cell surface receptors, that can be used as sensitive targeting agents. The goal of this review article is to explore molecular targets for HNSCC and dissect previously conducted research in nuclear medicine and provide a possible path forward for the development of novel radiopharmaceuticals used in targeted radionuclide therapy for HNSCC, which has been underexplored to date.

## 1 Introduction

The incidence of head and neck cancers (HNC) has risen globally in recent decades and is projected to continue to rise (1, 2). Head and neck squamous cell carcinoma (HNSCC) makes up more than 90% of head and neck cancers (3–6). HNSCC is a type of cancer that grows in the oral cavity, pharynx, nasal cavity, paranasal sinuses, salivary glands, and larynx (7, 8). Increasing cases of HNC have been linked to human papillomavirus (HPV) infection (1, 9, 10). Although HPV status has been determined to be an important factor in HNSCC diagnosis and prognosis, this review will not focus on HPV as it pertains to HNC. The current standard of care requires a multidisciplinary approach that combines surgical resection, radiation therapy, and adjuvant chemotherapy, of which most are associated with significant acute and long‐term toxicities (11). Despite advances in treatment, HNSCCs are associated with significant morbidity and mortality, with the 5-year overall survival rate of patients ranging from 30% to 65% (12). The substantial morbidity and mortality rates for HNSCC and the toxicity associated with the standard treatment options emphasize the need to seek alternatives. Nuclear medicine imaging and therapy techniques may be considered more tolerable than traditional chemotherapy because of their ability to specifically target cancer cells without causing harm to surrounding healthy tissue. This method may lead to better outcomes, including improved quality of life for patients.

Although radiation therapy has been deemed a standard option for the treatment of HNSCC, combining the use of monoclonal antibodies (mAbs) with radiotherapy, or *radioimmunotherapy*, has been of interest to amplify the effect of mAbs and trigger a strong antitumor immune response (13, 14). Accumulating evidence suggests that radioimmunotherapy can provoke tumor response through direct and immunogenic killing of the tumor cells, and this method is being investigated in numerous clinical trials (NCT03811015, NCT03452137, NCT02586207, NCT02707588, etc.) (15–17). In the early 1990s, the first radiolabeled mAb, ^111^Indium satumomab pendetide, was approved by the US Food and Drug Administration (FDA) for diagnostic imaging of colorectal and ovarian cancers, which helped pave the way for the use of radiolabeled antibodies to image and treat diseases (18). Although several mAbs alone have been approved for treatment of HNSCC, limited clinical trials have involved radiolabeled antibodies for imaging or therapy (19, 20).

Unlike traditional radiotherapy in which photons from an external source are directed at a tumor, damaging not only the cancerous cells, but also the healthy cells in the surrounding area, targeted radionuclide therapy (TRT) uses ionizing radiation to kill cells in a selective manner. TRT tethers a radionuclide to a targeting agent that delivers the radionuclide and its DNA-damaging particle emissions to a specific molecular target that is, typically, on the surface of the malignant cell. Radionuclides that decay via the emission of beta particles, alpha particles, or Auger electrons can be used to damage, and ultimately kill cancerous cells (21). By delivering the radionuclide directly to the tumor, fewer healthy cells will be damaged during treatment. The development of TRT for HNSCC may greatly improve patient outcomes.

Advancing TRT in HNSCC relies on understanding the mechanisms involved in the progression and development of this type of cancer. The epidermal growth factor receptor (EGFR) family, a group of molecular targets including EGFR (also known as human epidermal growth factor receptor 1, [HER1]), HER2, HER3, and HER4 play a role in the growth and survival of cancer cells (22). Currently, the only FDA-approved EGFR targeting mAb for HNSCC is cetuximab (23). However, due to recent advances in understanding molecular biology of HNSCC, it is reasonable to believe that targeted therapies using other molecular targets such as HER2 and HER3 that have been approved by the FDA for treatment of other cancers such as breast cancer, metastatic gastric cancers, and metastatic non-small cell lung cancer could be investigated for applicability to HNSCC (24–26). In addition to studying other EGFR targets for HNSCC treatment, targets such as programmed cell death protein 1 (PD-1) and programmed death-ligand 1 (PD-L1) could be of interest. In 2016, the FDA granted approval for pembrolizumab and nivolumab, which bind to PD-1 receptors for patients with recurrent or metastatic HNSCC (27). The development of novel diagnostic procedures and treatment methods such as TRT is paramount for the improvement of patient’s prognoses and quality of life, especially in HNSCC because of the complex nature of everyday functions within the head and neck area (7). This review article aims to discuss current treatment options for patients with HNSCC and to present a discussion on molecular targets that may be implicated in HNSCC, as well as how radiopharmaceutical development in this area could play a role in improved patient outcomes. This review outlines several molecular targets, beginning with the most widely studied EGFR. There are many molecular targets that exhibit potential in future development of radiopharmaceuticals, some of which are not exclusive to HNSCC.

## 2 Current treatment options

A multidisciplinary approach involving medical oncology, surgical oncology, radiation oncology, pathology, and radiology is required along with follow-up care, which may include physical or occupational therapy, speech or swallow therapy, and nutritional counseling to improve a patient’s quality of life (28). The current standard of care in HNSCC is either surgical resection followed by radiation therapy for early-stage disease or a combined administration of both chemotherapy and radiation for organ preservation, reserving surgery for residual disease (28–30). Cetuximab in combination with radiotherapy or platinum-based chemotherapy is the standard first-line therapy given for locally advanced, recurrent, or metastatic HNSCC (31–33). For individuals with recurrent disease not amenable to surgical resection or with metastasis, chemotherapy (cisplatin, 5-fluorouracil, or carboplatin) with or without immunotherapy is indicated for palliative treatment (30). In recent years, immunotherapy has emerged as a new treatment option that bolsters a person’s own immune system to help kill cancer cells (34). For patients who are candidates for immune checkpoint inhibitors (ICIs), pembrolizumab and nivolumab have been approved for the treatment of patients with recurrent or metastatic HNSCC with disease progression (32, 33, 35, 36). Candidates for ICIs are patients whose tumors express PD-L1 with a combined positive score ≥1 as determined by an FDA-approved test (PD-L1 IHC 22C3 pharmDx kit) (20). Although pembrolizumab, nivolumab, and toripalimab-tpzi are all FDA-approved targeting agents for HNSCC, no FDA-approved radiopharmaceuticals exist for the diagnosis or treatment of HNSCC.

## 3 Molecular targets

**Figure 1:**
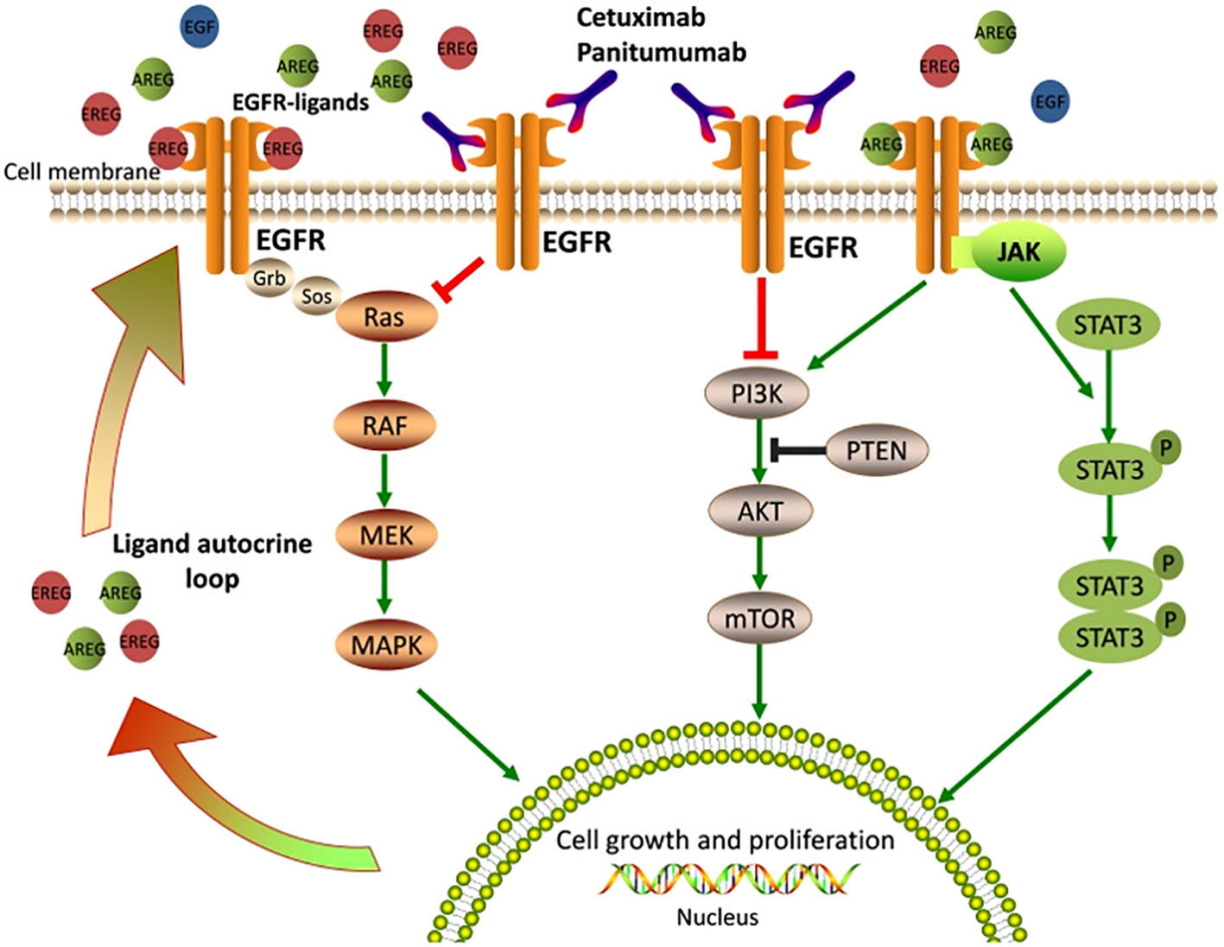
Epidermal Growth Factor Receptor signaling pathways and mechanisms of anti-EGFR therapy (42). EGFR ligands bind the extracellular domain of EGFR, leading to receptor activation and stimulating downstream signaling pathways that are crucial for cell growth and proliferation. Cetuximab or Panitumumab prevents ligand binding to EGFR, thus blocking EGFR signaling and inhibiting cell growth and proliferation (42).

HNSCC has multiple molecular alterations contributing to the development and progression of disease and numerous oncogenic targets that hold promise for targeted therapy have been characterized (37). High EGFR expression stands out, especially in HPV-associated HNSCC, which is an increasingly prominent risk factor of this disease (37). Four different receptors are in the Erb-b2 receptor tyrosine kinase (ERBB) family: EGFR (HER1), HER2, HER3, and HER4. Among the molecular targets that have been identified in HNSCC, HER2 and HER3 have emerged as promising therapeutic targets (38). EGFR can be activated either by binding to one of its ligands (epidermal growth factor [EGF], transforming growth factor-α [TGF- α], and amphiregulin) or by dimerization with other HER family receptors, specifically favoring HER2 (39). Dimerization with other HER family members leads to EGFR tyrosine kinase activity followed by downstream phosphorylation of key tyrosine residues in the receptor (7). These residues act as binding sites for intracellular signaling, leading to the activation of EGFR-mediated signaling pathways (7). Activating EGFR results in downstream cascade effects such as proliferation and pro-survival intracellular signaling through the mitogen-activated protein kinases (MAPKs) cascade, phosphatidylinositol-3-kinase (PI3K)/AKT/mammalian target of rapamycin (mTOR) and Janus Kinase (JAK)/signal transducer and activator of transcription (STAT) pathway (39–41). These downstream signaling pathways are depicted in **Figure 1**, which is adapted from Zhao et al. (42).

Preclinical and clinical studies have demonstrated the efficacy of EGFR-targeted therapies, such as cetuximab and panitumumab, in improving clinical outcomes in HNSCC patients (43–45). Additionally, emerging evidence suggests that targeting HER2 and HER3 may also be effective in HNSCC treatment (24). While numerous targets and molecular pathways relevant to HNSCC have been identified, most are not exclusive to HNSCC. This review aims to summarize the molecular pathways and targets that have been identified for use in TRT of HNSCC. **Table 1** lists the antibodies that are the focus of this review as well as the respective molecular targets, while it should be noted that there are indeed other prospective molecular markers that could be studied for HNSCC targeted radiopharmaceutical development (i.e, Pcl-XL, Cyclin D1, PI3K mTOR, CDK4/6, amongst others). Although numerous antibodies relevant to HNSCC have been identified, few have been radiolabeled for use in TRT. To develop radiopharmaceuticals leveraging these characteristics, antibodies can be incubated with a bifunctional chelator under mild conditions to produce an antibody drug-conjugate. The resulting conjugate can be radiolabeled to produce a radiolabeled mAb that can be administered *in vivo* for selective delivery to the tumor cells, therefore, demonstrating a promising targeted treatment method for HNSCC.

**Table 1:** Antibodies and respective targeting agent(s).

Antibody	Immunotherapeutic Strategy	Molecular Target	FDA Approval For HNSCC	References
Cetuximab	Humanized mAb	EGFR	Yes	(23, 34, 45–47)
Gefitinib	TKI	EGFR	No	(6, 48)
Erlotinib	TKI	EGFR	No	(6, 49)
Panitumumab	a fully human, IgG2 anti-EGFR mAb	EGFR	No	(50)
Afatinib	TKI	EGFR/HER2	No	(51)
Lapatinib	TKI	EGFR/HER2	No	(52–57)
Dacomitinib	TKI	EGFR/HER2	No	(26, 58)
AC480	PanHER inhibitor	EGFR/HER2	No	(59)
Trastuzumab	mAb	HER2	No	(25, 60)
Poziotinib	TKI	EGFR/HER2	No	(61, 62)
Pertuzumab	mAb	HER2	No	(60)
Patritumab	fully humanized IgG1 mAb	HER3	No	(63)
Seribantumab	fully humanized IgG2 mAb	HER2/HER3	No	(63)
Lumretuzumab	glycoengineered humanized mAb	HER3	No	(64)
Pembrolizumab	ICI mAb	PD-1	Yes	(27)
Nivolumab	ICI mAb	PD-1	Yes	(50)
Durvalumab	fully human IgG1 mAb	PD-L1	No	(65, 66)
Duligotuzumab	Dual-action humanized IgG1 mAb	EGFR/HER3	No	(24, 67, 68)
ISU104	Fully human anti-HER3 antibody	HER3	No	(63, 69, 70)
CDX-3379	Human mAb	HER3	No	(63, 71, 72)
AV-203	Humanized IgG1 mAb	HER3	No	(63, 73–75)
REGN1400	Fully human IgG mAb	HER3	No	(63, 76, 77)
Sym013	Mixture of 6 mAbs (3 pairs with each targeting EGFR, HER2 and HER3)	EGFR/HER2/HER3	No	(63, 78)
IgG 3–43	Human antibody	HER3	No	(63, 79, 80)
cmAb U36	Chimeric mAb	CD44v6	No	(81–84)

HNC, head and neck cancer; HNSCC, head and neck squamous cell carcinoma; HPV, human papillomavirus; mAb, monoclonal antibody; FDA, food and drug administration; TRT, targeted radionuclide therapy; EGFR, epidermal growth factor receptor; HER2, human epidermal growth factor receptor 2; HER3, human epidermal growth factor receptor 3; PD-1, programmed cell death protein 1; PD-L1, programmed death-ligand 1; ICI, immune checkpoint inhibitor; EGF, epidermal growth factor; TGF- α, transforming growth factor-α; MAPK, mitogen-activated protein kinase; PI3K, phosphatidylinositol-3-kinase; mTOR, mammalian target of rapamycin; JAK, janus kinase; STAT, signal transducer and activator of transcription; Ig, immunoglobulin; TKI, tyrosine kinase inhibitor; NRG, neuroregulin; SPECT, single photon emission computed tomography; PET, positron emission tomography; CT, computed tomography; ^18^F-FDG, ^18^F-fluorodeoxyglucose; LN, lymph node; SUV_max_, maximum standard uptake value; LET, linear energy transfer; TKI, tyrosine kinase inhibitor; HB-EGF, heparin-binding epidermal growth factor; TGF, tumor growth factor.

### 3.1 Epidermal growth factor receptor

EGFR is expressed in healthy tissues throughout the body and plays a role in cell growth, proliferation, and differentiation (85). EGFR overexpression has been implicated in several types of cancer such as colorectal, breast, ovarian, pancreatic, bladder, and HNSCC (85–87). EGFR is overexpressed in up to 90% of HNSCC cases and is associated with poor prognosis as well as resistance to chemotherapy and radiation therapy (31, 88). EGFR-targeted therapies incorporating mAbs and tyrosine kinase inhibitors have been developed and demonstrate antitumor activity.

#### 3.1.1 EGFR-targeted therapy using antibodies

Studies found that in combination with chemotherapy or radiation therapy, cetuximab showed a clinical benefit in the treatment of HNSCC, and the drug was approved by the FDA in 2006 (89, 90). In a review paper published in November of 2019, Gougis et al. stated the only known targeted therapy that has shown an overall survival benefit to HNSCC is cetuximab (46). Panitumumab is an immunoglobulin (Ig) G2 mAb that blocks EGFR; IgG2 mAb is FDA-approved for the treatment of patients with EGFR-expressing, metastatic colorectal cancer (42). The benefit of panitumumab in these patients has led to the translation of further studies for use in patients with EGFR-expressing HNSCC. In a phase 2 trial of 46 high-risk, resected, HPV-negative HNSCC patients, Fasano et al. targeted the EGFR pathway using panitumumab in combination with cisplatin chemoradiotherapy (39). This study concluded that panitumumab in combination with cisplatin chemoradiotherapy is well-tolerated and demonstrates improved clinical outcome when compared with historical controls with standard treatment (39). In a phase III trial (SPECTRUM), the efficacy and safety of panitumumab combined with cisplatin and fluorouracil as a first line treatment for recurrent or metastatic HNSCC was assessed. A retrospective analysis was completed to assess tumor HVP status as a predictive biomarker with p16-INK4A (p16). The study included 657 patients, in which 330 patients received chemotherapy only and 327 patients received chemotherapy plus panitumumab. There was found to be no significant improvement of median overall survival; however, the median progression-free survival for p16-negative patients was improved for the panitumumab plus chemotherapy group (91).

Notably, resistance to cetuximab and panitumumab is common, and several mechanisms of resistance have been identified, including EGFR mutations and activation of alternative signaling pathways. Alterations in the RAS-RAF-MAPK, PI3K-PTEN-AKT, and JAK/STAT pathways activated by EGFR such as KRAS, NRAS, BRAF, and PIK3CA gene mutations, can lead to downstream signaling cascades from EGFR activation, leading to drug resistance (42, 92, 93). Genetic alterations, including RAS, BRAF, PIK3CA, EGFR S492R mutations, PTEN loss, and STAT3 phosphorylation in the members of EGFR signaling pathways, contribute to the resistance through EGFR activation downstream signaling cascades (42).

#### 3.1.2 EGFR-targeted therapy using small molecules

Although trials assessing the benefits of EGFR inhibitors in HNSCC patients have been published, the benefits of tyrosine kinase inhibitors (TKIs), such as gefitinib and erlotinib, in advanced HNSCC are still unknown (48). Tang et al. analyzed seven randomized controlled clinical trials and assessed the effectiveness and safety of gefitinib-based therapy in patients with advanced HNSCC in comparison with standard regimens. The study found that the benefits from gefitinib-containing regimens cannot prolong the overall survival or progression free survival or improve overall response rate; however, for recurrent patients, gefitinib tends to improve quality of life (48). According to the review article authored by Byeon et al., gefitinib and erlotinib, which target only EGFR, have not had clinical benefit in HNC; however, afatinib, lapatinib and dacomitinib, which are multitarget TKIs that target EGFR and HER2, have shown potential in clinical trials (26, 49, 51, 52, 58). These multitarget TKIs are discussed further in section 3.2.2. Another phase II study assessed the antitumor activity of afatinib in comparison to cetuximab in 121 patients with recurrent or metastatic HNSCC. Afatinib showed more antitumor activity than cetuximab, however, patients administered afatinib had more intolerable adverse events (51).

### 3.2 Human epidermal growth factor receptor 2

**Figure 2:**
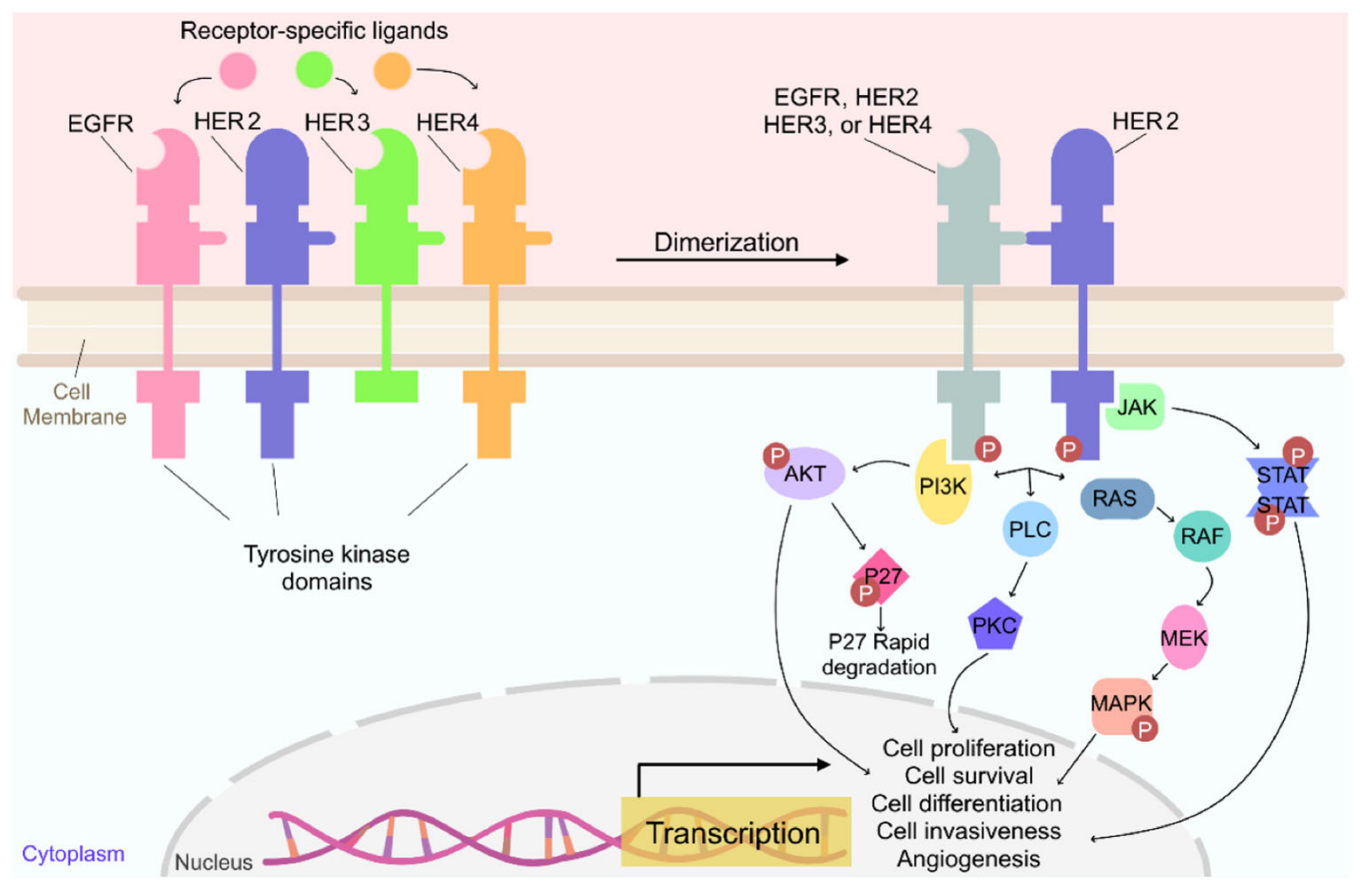
Overview of the signaling pathway for HER2 (97).

HER2 is a type of EGFR that is overexpressed by some types of breast cancers and other types of cancer such as ovary, endometrium, bladder, lung, and colon. Pollock et al. found that HER2 is overexpressed in the range of 0% to 47% of HNSCC’s and suggested that targeting HER2 may be effective in treating some HNSCC’s (40, 94–96). Although the EGFR family of receptors are mainly activated through ligand binding to the extracellular domain, HER2 does not have a natural ligand and becomes activated upon heterodimerization with another activated EGFR family of receptors or homodimerization with another HER2 receptor (**Figure 2**) (40, 97–100). HER2 activation leads to subsequent activation of downstream signaling pathways such as PI3K/AKT, RAS/MEK/MAPK, JAK/STAT, and PKC, in turn promoting cell proliferation, survival, differentiation, angiogenesis, and invasion (40, 97–100). Activated PI3K/AKT also leads to the degradation of cell-cycle inhibitor p27Kip1, promoting cell-cycle progression (97).

#### 3.2.1 HER2-targeted therapy using antibodies

Several HER2-targeted therapies have been developed, including mAbs, such as trastuzumab and pertuzumab (25, 60). These therapeutic agents can block HER2 signaling, either by inhibiting its kinase activity or by preventing its interaction with other EGFR family members. Trastuzumab is a humanized mAb that blocks HER2 signaling and prevents HER2 dimerization and intracellular tyrosine kinase activation (101). HER2-targeted therapies using trastuzumab have shown clinical benefit in HER2-positive breast cancer and gastric cancer and have been approved by the FDA (102, 103). While not yet approved for HNSCC, numerous clinical trials have investigated the clinical use of trastuzumab to target HER2 in HNSCC. A phase 2 study of trastuzumab in 14 patients with advanced salivary gland tumors overexpressing HER2 reported that given as a single agent, trastuzumab had low activity and did not provide clinical benefit (104). Another phase 2 study conducted by Gillison et al. evaluated the response rate of HNSCC patients to trastuzumab in metastatic or recurrent HNSCC based on HER2 expression by immunohistochemistry. The authors concluded that trastuzumab did not improve the response rate in comparison to the standard of care cisplatin or paclitaxel and attributed the ineffectiveness to the lack of HER2 expression (25).

The use of the anti-HER2-antibodies or HER2 inhibitors in combination with irradiation have shown promise and could potentially be a new path forward for treating HNSCC. A study evaluating the effects of the combination of recombinant humanized monoclonal antibody (rhumAb)HER2, an anti-HER2 antibody, with 4 Gy irradiation by x-rays demonstrated that combined treatment enhanced growth inhibition effects in HNSCC cell lines when compared with rhumAbHER2 or irradiation alone (105). While this is promising *in vitro* data, further research should be conducted to verify the growth inhibition effects *in vivo*.

#### 3.2.2 HER2-targeted therapy using small molecules

Afatinib is an irreversible kinase inhibitor that targets both EGFR and HER2. In a preclinical trial administering afatinib, a human HNSCC cell line with an overexpression of HER2 was used to generate cetuximab-resistant clones from a cetuximab-sensitive bladder cancer cell line *in vivo*. Cetuximab-sensitive xenografts were exposed to increasing concentrations of cetuximab followed by validation of the resistant phenotype *in vivo* and *in vitro* using invasion assays. Cells were injected into 40 mice and then randomly distributed into four treatment groups with 10 mice per group. Each group was treated with cetuximab, afatinib, or both. The study showed afatinib inhibited growth of the cetuximab-resistant cells *in vitro* and increased growth inhibition in the cetuximab-resistant xenografts when combined with cetuximab *in vivo* (106).

Lapatinib is a dual inhibitor of both EGFR and HER2 tyrosine kinases that acts by blocking growth signals, which stops the cell from growing and dividing. When used with cisplatin, the efficacy was further increased, suggesting that lapatinib heightens the sensitivity of HNSCC towards cisplatin (53). Schrader et al. studied the combination therapy of lapatinib and cisplatin and its effects on colony formation of epithelial cells in HNSCC; the study concluded that lapatinib inhibits colony formation of epithelial HNSCC cells (53). Inhibition of tumor colony formation holds significance in assessing therapeutic effectiveness because the ability to impede the development of colony formations carries the potential to slow the advancement of the cancer. Kondo et al. looked at the antitumor effects of lapatinib in combination therapy with cisplatin or paclitaxel on HNSCC lines both *in vitro* and *in vivo*; the study found that lapatinib did not significantly inhibit angiogenesis. However, combination treatment between lapatinib and chemotherapy further increased the efficacy of antitumor activity by increasing apoptosis, and the study surmised that lapatinib might provide a better treatment path for HNSCC patients (54). Furthermore, a 2007 study assessed the ability of a statistical model to analyze protein expression levels of EGFR and HER2 in response to lapatinib in 61 different human tumor cell lines from 12 tumor types, two oncogene transformed human cell lines, and two normal human cell cultures. The study found that applying HPV status as a parameter along with the expression of EGFR and HER2 improved the statistical model’s ability to predict lapatinib sensitivity (55). Computational models can help suggest causes and correlative factors in cancer that could provide insight into tumor response and cancer progression during treatment.

In a randomized phase 2 study, the effects of lapatinib monotherapy were measured on 107 therapy-naive patients presenting with locally advanced HNSCC. The study concluded that short term lapatinib monotherapy did not demonstrate statistically significant apoptotic changes but provided evidence of clinical activity (56). In another phase 2 clinical study performed by De Souza et al. to determine the efficacy and safety profile of lapatinib in patients with recurrent or metastatic HNSCC, the results suggested that lapatinib as a single agent was well-tolerated but was a weak EGFR inhibitor and lacked efficacy (57). Another randomized phase 2 study of oral lapatinib plus chemotherapy in patients with locally advanced HNSCC assessed the activity and safety of concurrent chemoradiotherapy. Sixty-seven patients were randomly assigned to concurrent chemoradiotherapy plus placebo or concurrent chemoradiotherapy plus lapatinib. The progression-free survival and overall survival rates at 18 months were determined to be 55% versus 41% for the placebo group compared to 68% and 57% for the group administered lapatinib, leading to the conclusion that lapatinib combined with chemoradiotherapy was well-tolerated with numeric increases in the complete response rates (52).

Poziotinib, a dual inhibitor of EGFR and HER2, has shown efficacy against various types of cancers in phase 1 studies (61). In a phase 2 study conducted with 49 patients with a median age of 62 years, Lee et al. concluded that poziotinib showed clinically meaningful efficacy with manageable toxicity in patients with heavily treated recurrent and/or metastatic HNSCC (61). Based on these results, Li et al. drew the conclusion that poziotinib therapy was “noninferior” to afatinib monotherapy in a review published in January 2023 (62). In a review conducted in October 2022, Liu et al. noted that poziotinib tested in patients with HER2‐positive tumor types such as gastric cancer, breast cancer, and HNSCC yielded promising antitumor efficacy with manageable toxicity (61, 107–109).

AC480, a novel EGFR and HER2 inhibitor, administered before and during exposure to gamma radiation, was discovered to enhance the radiosensitivity of HNSCC cells and significantly reduce tumor size *in vivo* (59).

Although small molecules and antibodies have been studied for treatment mechanisms against HNSCC, a gap in research has yet to be filled for the strategic use of HER2 as a molecular target for TRT. There is an urgent need for more clinical evidence to support the role of HER2 targeting in HNSCC.

### 3.3 Human epidermal growth factor receptor 3

Another member of the EGFR family is human epidermal growth factor receptor 3 (HER3). Some studies have indicated HER3 ligand neuregulin 1 (NRG) is expressed highly in HNSCC and is correlated with poor prognosis (110–112). Clinical trials are ongoing to evaluate the efficacy of HER3-targeted therapies, such as mAbs and tyrosine kinase inhibitors, in HNSCC treatment (24).

A phase 1b study investigated the clinical efficacy and safety of duligotuzumab, a dual action humanized IgG1 mAb that targets EGFR and HER3, in combination with chemotherapy in the treatment of patients with recurrent or metastatic HNSCC. The authors concluded that the addition of duligotuzumab revealed a promising response rate of 67% but witnessed an increased frequency of adverse events in patients (113). A 2022 review paper published by Gandullo-Sánchez et al. reported ISU104, a fully human anti-HER3 antibody, showed more than 70% tumor growth inhibition in HNSCC in the studies they reviewed (113). Additionally, the HER3-targeting human antibody, IgG 3–43, exhibited efficacy in FaDu HNSCC murine xenograft models (24, 67). In a window-of-opportunity study, Duvvuri et al. concluded CDX-3379, a human mAb, alone or in combination with cetuximab was well tolerated and caused tumor regression in HNSCC (63, 69, 70, 79, 80). AV-203, a humanized IgG1/HER3 inhibitory antibody, has also been reported to inhibit tumor growth in HNC and esophageal cancer models. Additionally, the authors concluded that NRG1 can be used to predict response to HER3 inhibition by AV-203 (63, 69, 70, 79, 80). Activation of HER3 is controlled by the availability of NRG, but HER3 can also be activated by HER2 in tumors that highly overexpress HER2 (63, 71, 72). To gain further insight into HER3 signaling, REGN1400 (a potent blocking antibody against HER3) was administered alone and in combination with the EGFR blocking antibody REGN955. Both agents were found to slow tumor growth as a single agent but had increased efficacy in combination with the EGFR blocking antibody. The results from this study suggest that EGFR and HER3 are coactivated in many cell lines, and the combined blockade of both receptors inhibits cell growth more effectively (76). A first-in-human phase 1 study for patients with metastatic or advanced HER3-positive carcinomas showed that lumretuzumab, a glycoengineered humanized mAb, was well-tolerated and reported signs of clinical activity (114).

Patritumab, a fully human IgG1 mAb that inhibits ligand binding to HER3, was studied in untreated recurrent and/or metastatic HNSCC (24, 115). Forster et al. conducted a randomized phase 2 study in which 87 patients were assigned to receive patritumab or placebo, both in combination with cetuximab and up to six cycles of platinum therapy, and concluded that no statistically significant improvement occurred in progression-free survival or overall survival, with the trend favoring the placebo group in overall survival (24, 115). Although patritumab has been shown to have limited activity, patritumab deruxtecan, an antibody–drug conjugate, has shown encouraging response rates in studies and trials for other HER3 positive cancers and is now being extended to HNSCC (24, 113, 116, 117).

Sym013 (Pan-HER) is a mixture of six mAbs that comprises 3 pairs of mAbs, each targeting EGFR, HER2, and HER3 yielding simultaneous inhibition of all three receptors. Sim013 has been reported to effectively inhibit the growth of lung and HNSCC cancer models *in vitro* and *in vivo* (118, 119). Sym013 causes degradation of EGFR, HER2, and HER3, preventing ligand binding to EGFR and HER3, and inhibiting activation of the AKT and MAPK/ERK pathways (118). Francis et al. demonstrated that Pan-HER combined with single or fractionated radiation showed a potent antitumor effect and delayed regrowth in HNSCC xenografts, including cetuximab-resistant models (78). Further understanding of the molecular mechanisms involved in HER3 signaling and its interactions with other EGFR family members may facilitate the development of effective HER3-targeted therapies for HNSCC. Targeting HER3, either alone or in combination with other EGFR family members could be a promising strategy for HNSCC treatment or may pose a potential pathway to overcome HER and HER2 resistance mechanisms seen in some HNSCC cases. The full potential of HER3 targeting is still unknown and remains to be investigated through further preclinical and clinical trials.

## 4 Other molecular targets

Immunotherapy leveraging ICIs is a more recent treatment option under investigation for advanced HNSCC. ICIs block checkpoint pathways in the cell by preventing checkpoint proteins on T-cells from binding to similar proteins on tumor cells, which bolsters the immune cells and restores the antitumor immune responses that promote the elimination of cancer cells (120). Currently, the main ICIs for immunotherapy that have been used in the treatment of HNSCC target PD-1 receptors and PD-L1 receptors (33, 120, 121).

Pembrolizumab is a humanized mAb that binds to PD‐1 and blocks its interaction with PD‐L1 (27). The FDA granted accelerated approval in August 2016 for pembrolizumab for treatment of patients with recurrent or metastatic HNSCC with disease progression on or after platinum-based chemotherapy in August 2016, making it the first drug to receive approval for the treatment of patients with HNSCC since cetuximab was approved for this indication in 2006 (27). Approval was based on the objective response rate and duration of response in patients in a nonrandomized multi-cohort trial conducted by Larkens et al. (27). In a randomized trial, Ferris et al. found that nivolumab, a mAb that binds to PD-1, had fewer toxic effects compared with standard therapy (chemotherapy or cetuximab) and prolonged survival rates in patients that had disease progression after platinum-based chemotherapy treatment in HNSCC (50). Based on the data from this clinical trial, the FDA approved nivolumab in November 2016 (50, 122).

Durvalumab is a mAb that binds to PD-L1 receptors (65). In a phase 1/2 study which included 62 patients with recurrent or metastatic HNSCC, Segal et al. evaluated durvalumab monotherapy and achieved an objective response rate of 11% (123, 124). In the phase 2 study, which included 112 patients with recurrent or metastatic HNSCC that had progressed after one line of platinum-based therapy, Zandberg et al. demonstrated durvalumab monotherapy treatment had an objective response rate of 16.2% with a median duration of response of 10.3 months in patients with 25% or more tumor cells expressing PD-L1 (123–125). Fujiwara et al. initiated a preliminary dose escalation phase designed to assess the safety, tolerability, and pharmacokinetics of escalating doses and different dosing schedules of durvalumab monotherapy; their study determined that durvalumab at the doses and regimens evaluated was safe and well-tolerated in Japanese patients with advanced solid tumors (66, 126). Doki et al. found durvalumab monotherapy demonstrated acceptable safety profiles and clinical benefit in a phase 1 study for patients from Asia with HNSCC who had progressed on previous systemic chemotherapy (66). However, their study noted that of the 32 patients with HNSCC, 19 of the patients had a PD-Ll expression ≥ 1% and only 8 patients had a PD-Ll expression ≥ 25% (66).

Fibroblast activation protein (FAP) is a cell surface serine protease that has been identified as a biomarker of cancer-associated fibroblasts (127). FAP is highly expressed in more than 90% of epithelial tumors and is nearly undetectable in healthy tissue, making it a potential target for HNSCC (128). Fibroblast activated protein inhibitors (FAPIs) have been developed to provide a basis for FAP targeting radiopharmaceuticals and will be discussed further in the next section.

CD44v6 is an oncogenic variant of the cell surface molecule CD44 that is overexpressed in HNSCC, lung, skin, esophageal, and cervical cancers (129). CD44v6 overexpression has been observed in more than 90% of primary and metastatic HNSCC and is associated with disease progression and radioresistance, making it an attractive molecular target (81–83).

## 5 Radiolabeled antibodies

The use of radiopharmaceuticals for the treatment of many types of cancer is an active area of research. However, development of radiopharmaceuticals for HNSCC is still lacking. Clinical studies on TRT for HNSCC are limited likely because of the small amount of preclinical research. This section aims to cover the breadth of research in radiolabeled antibodies for diagnostic imaging and TRT for HNSCC, but the research focus in this area is small to date. Diagnostic imaging agent development is a crucial step in determining potential TRT analogs. Although brief, several diagnostic imaging radiopharmaceuticals are discussed herein, followed by subsequent studies discussing therapeutic radiopharmaceuticals that leverage properties of alpha- or beta-emitting radionuclides. Either alone or in combination with conventional therapies, radiopharmaceuticals can improve the therapeutic efficacy of mAbs (130–132). Radionuclides including ^64^Cu, ^68^Ga, and ^89^Zr have been used to radiolabel mAbs for diagnostic imaging. Radionuclides including ^90^Y, ^131^I, ^177^Lu, ^188^Re, and ^225^Ac have used to radiolabel mAbs for TRT. These radionuclides have been evaluated in several HNSCC studies and will be discussed further in the sections below.

### 5.1 Radiopharmaceuticals for imaging

Radiopharmaceuticals for diagnostic imaging make use of target-specific molecules, while leveraging the nuclear properties of these radionuclides to obtain high resolution images, giving insight into disease status and extent. ^89^Zr-Df-nivolumab has been used to image the biodistribution of PD-1 expressing tumors in a humanized murine model of lung cancer. The tracer demonstrated high binding to stimulated PD-1-expressing T-cells *in vitro* and *in vivo* (133). This study concluded that the positron emission tomography (PET) resolution was great enough to support their claim of PD-1 being a good target for tumor imaging *in vivo* as well as the possibility as a target for therapy (130, 134, 135). Although this study focused on imaging lung cancer, it might prove beneficial for other cancers, such as cases of HNSCC that express PD-1, as well (136).

To identify patients who may benefit from EGFR targeted therapy, an ^111^In-labeled cetuximab tracer was developed for single photon emission computed tomography (SPECT) imaging of nude mice bearing FaDu HNSCC xenografts. Tumor uptake was favorable and correlated well with intratumoral distribution determined by autoradiography and EGFR immunohistochemistry. However, tumor accumulation and blood clearance rates were low. The tumor-to-background contrast increased with time requiring later imaging timepoints, and therefore made the tracer less suitable for clinical applications (137). In a follow-up study, the authors developed and characterized an ^111^In-cetuximab-F(ab’)_2_ tracer using the same model and found that ^111^In-cetuximab-F(ab’)_2_ displayed higher tumor-to-blood ratios making it more suitable for EGFR visualization (137, 138). ^89^Zr-cetuximab was developed for *in vitro* and *in vivo* studies using tumor cell lines with varying EGFR expression levels. *In vitro* studies demonstrated EGFR uptake; however, a disparity was shown between antibody uptake and expression levels *in vivo* indicating that EGFR expression levels alone are not sufficient for predicting patient response (139). Cetuximab resistance poses a problem for patients with HNSCC, resulting in increased tumor recurrence rates and showing limited clinical efficacy. Cetuximab resistance in HNSCC was investigated using ^89^Zr-cetuximab, and the study found that it was a suitable radiopharmaceutical to identify EGFR downregulation *in vitro* and *in vivo*, demonstrating the potential applicability for monitoring patient resistance during cetuximab therapy (140). A phase 1 trial to determine the safety of ^89^Zr-cetuximab assessed tumor uptake in HNC and non-small cell lung cancer patients and determined that no additional toxicity was associated with its administration in comparison with unlabeled cetuximab (141). Studies recommend imaging 6 to 7 days after ^89^Zr-cetuximab injection, which is approximately two half-lives of the radionuclide used (^89^Zr) (141, 142).

^64^Cu-panitumumab was implemented to acquire small-animal positron emission tomography (PET) images in mice bearing human HNSCC xenograft tumors, and the *in vivo* kinetics of antibody was visualized (143). The study determined that no correlation exists between PET resolution and EGFR protein expression level. The tumors with the lowest EGFR expression had the highest distribution of the tracer, and the highest EGFR expression had the lowest tracer distribution (143). In a clinical study, the safety and specificity of ^89^Zr-panitumumab for diagnosis of HNSCC in patients was studied and compared with ^18^F-fluorodeoxyglucose (^18^F-FDG) PET/computed tomography (CT). The combined ability of ^18^F-FDG PET/CT and ^89^Zr-panitumumab PET/CT to detect HNSCC was improved with a specificity of 96.3% in comparison with 74.1% with ^18^F-FDG PET/CT alone. The authors concluded that ^89^Zr-panitumumab PET/CT imaging is safe and may be of use for discriminating incidental findings from ^18^F-FDG PET/CT from true positive lesions (144). Current clinical trials using ^111^In-panitumumab are ongoing to evaluate the safety and effectiveness for identifying the first lymph nodes (LNs) to which cancer has spread from the primary tumor in patents with HNSCC undergoing surgery (NCT05901545).

The diagnostic imaging performance of a ^89^Zr-labeled-chimeric mAb U36, which targets CD44v6, was evaluated in patients with HNSCC who were determined to be at high risk of neck lymph node metastases. The tracer was able to detect all the primary tumors and 72% of positive levels, indicating that the tracer performed at least as good as CT/magnetic resonance imaging (MRI) for detection of metastases in HNSCC patients (145, 146).

Clinical studies have been conducted using radiolabeled FAPIs for PET imaging of primary and metastatic HNSCC. In a study aimed to compare the detection capability of ^68^Ga-FAPI-46 PET/CT compared with ^18^F-FDG PET/CT, the authors found that ^68^Ga-FAPI-46 PET/CT and ^18^F-FDG PET/CT demonstrate comparable diagnostic performance (147). Giesel et al. compared ^68^Ga-FAPI-2 and ^68^Ga-FAPI-4 to ^18^F-FDG and provided a preliminary dosimetry estimate. The authors found that the normal organs showed a low tracer uptake and the ^68^Ga-labeled FAPI tracers performed equally at 1-hour post-injection. The study concluded that the FAPI tracers provided tumor-to-background contrast ratios greater than or equal to those of ^18^F-FDG (148). A head-to-head comparison of ^68^Ga-FAPI-04 PET/CT to ^18^F-FDG PET/CT was conducted to compare the diagnostic value of ^68^Ga-FAPI-04 PET/CT and ^18^F-FDG PET/CT imaging for primary lesions and metastatic lymph nodes in 21 tonsil cancer patients. The study found that ^68^Ga-FAPI-04 PET/CT was more efficient in detecting primary lesions but had a lower maximum standard uptake value than ^18^F-FDG PET/CT. Additionally, ^68^Ga-FAPI-04 PET/CT demonstrated an improved distinction between the primary tumor and contralateral normal tonsil tissue. There was not a statistically significant difference in the maximum standard uptake value or the tumor-to-background ratio for lymph node analysis comparing ^68^Ga-FAPI-04 PET/CT to ^18^F-FDG PET/CT; however, the specificity and accuracy of ^68^Ga-FAPI-04 PET/CT was higher. The study concluded that ^68^Ga-FAPI-04 PET/CT is complementary to ^18^F-FDG PET/CT by improving the detection of primary lesions and the diagnostic accuracy of lymph node metastases in tonsil cancer (149).

### 5.2 Therapeutic radiopharmaceuticals

Therapeutic radionuclides can be classified into three broad categories, based on whether they emit alpha particles, beta particles, or Auger electrons. Alpha particle-emitting radionuclides are positively charged, heavy particles with significantly higher energies, very short path lengths, and have a high linear energy transfer (LET). Beta () particles are negatively charged electrons emitted from the nucleus with a long range and low LET, and Auger electron emitters emit electrons with a higher LET than beta particles but have a lower LET than alpha particles (130, 150, 151). A biomolecule (peptide, small molecule, monoclonal antibody, etc.) can be used to transport the radionuclide to the cell of interest. The antibody binds to its target, and the radionuclide then emits damaging ionizing radiation via its respective emission pathway during decay (130, 151). Radiolabeled antibodies rely on the decay characteristic of the employed radionuclide for therapy. Therefore, the notorious immune response that can be associated with unlabeled mAbs can be reduced because the radiolabeled antibodies do not rely on targeting signaling pathways to cause antitumor effects; they rely on the mAb to bring the radionuclide to the malignant cells, therefore less mAb can be injected (130, 152). Currently, no FDA-approved radiolabeled antibodies exist for the treatment of HNSCC; however, there are radiolabeled antibodies that are approved for use in clinical practice for other types of cancer that may be applied for treatment of HNSCC with similar molecular characteristics.

Parakh et al. provided an overview of the radiolabeled, intact antibodies currently in clinical use as well as those in development for cancers other than HNSCC. The study concluded that radioimmunotherapy demonstrated therapeutic efficacy in hematological cancers; however, the benefit of radiolabeled antibodies in the treatment of solid tumors has been less effective and several strategies are being investigated to improve their therapeutic index (130). Tumors with high antigen expression treated with fractionated radioimmunotherapy protocols (multiple doses rather than single dose) or radioimmunotherapy in combination with other agents, such as chemotherapy, have shown the best response (130, 133). Targeting patients with minimal residual disease using pre-targeting strategies and using newer radionuclides, combined therapeutic modalities, improved labeling techniques, and locoregional application are among the strategies proposed and being evaluated to improve the success rate of radioimmunotherapy (130, 132).

Few studies have been conducted using radiolabeled antibodies for HNSCC therapy. Preclinical studies investigated cell toxicity and cell binding properties of ^177^Lu-panitumumab and ^177^Lu-cetuximab using UM-SCC-22B tumor models. Both ^177^Lu-panitumumab and ^177^Lu-cetuximab exhibited favorable tumor targeting efficacy and significantly delayed tumor growth. However, ^177^Lu-panitumumab had a higher tumor uptake and more effectively inhibited tumor growth (130, 133). Another study looked at ^90^Y-cetuximab as a radioimmunotherapy agent. UM-SCC-22B and SCC1 HNSCC cells were xenografted in nude mice and PET imaged using ^18^F-FDG and ^64^Cu- cetuximab to monitor the therapeutic effect of ^90^Y- cetuximab. The authors found that UM-SCC-22B tumors, which had a lower EGFR expression, were more responsive to ^90^Y-cetuximab treatment than SCC1 tumors (130, 132). The combination of ^90^Y-cetuximab with fractionated radiotherapy was investigated in FaDu-tumor bearing mice. The mice were treated with fractionated radiotherapy alone, fractionated radiotherapy plus cold cetuximab, or fractionated radiotherapy plus ^90^Y-cetuximab and PET imaged using ^86^Y-cetuximab as a tracer. The authors found that low to moderate external beam doses can enhance antibody uptake, and the combination of ^90^Y-cetuximab and fractionated radiotherapy substantially increased tumor control compared to fractionated radiotherapy alone (153). The effects of cetuximab in combination with alpha-emitting radioimmunotherapy on a panel of HNSCC lines have been investigated. The cells were treated with cetuximab in combination with ^211^At-chimeric mAb (cmAb) U36, and the cell uptake, internalization, and cell proliferation were assessed. Cetuximab in combination with ^211^At-cmAb U36 mediated increased growth inhibition compared with radioimmunotherapy or cetuximab alone. However, cetuximab exerted radioprotective effects on the cell lines, suggesting interactions between CD44v6 and EGFR (136).

A phase 1 study investigated the safety, maximum tolerated dose, pharmacokinetics, dosimetry, immunogenicity, and therapeutic potential of a ^186^Re-labeled anti-CD44v6 chimeric mAb U36 (cmAb U36) in HNSCC patients. In total, 13 patients with recurrent or metastatic HNSCC were administered ^99m^Tc-cmAb U36 for planar and SPECT imaging, followed by ^186^Re-cmAb U36 one week later. The authors concluded that ^186^Re-cmAb U36 was well tolerated and could be safely administered with a dose-limiting myelotoxicity at 41 mCi/m^2^. Additionally, using ^99m^Tc-cmAb U36 to predict the pharmacokinetics of ^186^Re-cmAb U36 can provide insight into dose selection (154).

Proof-of-principle radioimmunotherapy studies conducted by Harris et al. have investigated the use of ^188^Re-labeled mAbs to treat HPV-related HNSCC by targeting E6 and E7 oncogenes (155, 156). Although beyond the scope of this review, we direct the interested reader to these studies.

Limited TRT studies have been done for HNSCC; however, there are new clinical trials underway including using ^225^Ac in TRT of HNSCC (NCT03746431) (157). The results of these clinical trials will hopefully prove to be an exciting addition to current treatment options for patients living with HNSCC.

## 6 Discussion and future directions

The identification of new molecular targets is crucial in developing radiopharmaceuticals for TRT of HNSCC. Precision medicine will help stratify patients based on their specific disease characteristics; therefore, predictive biomarkers and a deep understanding of the mechanisms occurring at the molecular level are needed in order to understand, and ultimately treat, all patients regardless of the cancer subtype. Thus far, research has focused on targeted pharmaceuticals using mAbs or ICIs; however, weak evidence exists of success because of monotherapies and drug resistance have been noted. The combination of targeting agents with other therapies can enhance treatment efficacies to achieve improved outcomes and overcome barriers associated with monotherapies. TRT has significant potential by bringing cytotoxic radionuclides directly to the site of disease, minimizing the need for conventional radiotherapy or chemotherapies that obliterate healthy cells in the process. Success in TRT relies on understanding the properties, expressions, and interactions of molecular targets.

The EGFR family is a promising target that is commonly overexpressed in HNSCC. The challenge of targeting EGFRs arises from the expression in many normal tissues. Anti-EGFR agents such as cetuximab targeting the extracellular domain of EGFR have already been FDA-approved but have demonstrated limited benefits in combination with other treatments. Although EGFR is overexpressed in more than 80% of HNSCC tumors, no clear predictive response exists with cetuximab efficacy. However, some studies have hypothesized cetuximab makes the cells more radiosensitive. The enhanced radiosensitivity exerted after cetuximab administration could be leveraged by radiolabeling cetuximab with therapeutic radionuclides and should be investigated for therapeutic efficacy in future studies. In addition to this, patients could see benefit from cetuximab radiolabeled with diagnostic PET or SPECT radionuclides which would provide information on receptor status, and in turn give insight as to whether patients would benefit from EGFR-targeted therapy. Stratifying patients based on receptor expression in early stages of their disease could certainly lead to improved prognosis (158, 159).

Tyrosine kinase inhibitors (TKIs) targeting the intracellular EGFR domain have shown clinical benefits in some cases, but a significant need still exists for more research to identify patients who may see a positive response with these therapies. Previous clinical studies involving TKIs have been conducted with broad patient populations, which limits the probability of success in the trials. This can be overcome by focusing on identification and analysis of biomarkers to select patients for these clinical trials that will most likely respond to TKI treatments. Thus far, no TKIs have been approved by the FDA for HNSCC treatment and further research is warranted to determine the clinical benefit. While EGFR-targeting TKIs have shown limited success, multitargeting TKIs such as afatinib or lapatinib have shown clinical potential and could be the focus of future radiolabeling experiments.

Few studies incorporate radiopharmaceuticals for the diagnosis or treatment of HNSCC; however, the number of successful clinical trials and FDA-approved radiopharmaceuticals in other types of cancers such as ^223^Ra dichloride (Xofigo) and ^177^Lu-DOTATOC (Lutathera) demonstrate the clinical efficacy of radiopharmaceuticals and suggest the potential use in HNSCC. The current use of TRT has already identified a number of targets that can be translated to the diagnosis and treatment of HNSCC and more continue to evolve with the increasing number of clinical studies.

Further identification of radiometals for use in the TRT radiopharmaceuticals should focus on the half-life, nuclear decay characteristics, and pricing and availability of the isotope. The isotope should have enough range and high-enough energies to effectively kill the tumor while limiting the radiation dose to the healthy tissue. The radionuclides should be compatible with the pharmacokinetics and the biological half-life of the targeting vector. The future of TRT is fueled by a strong understanding and optimization of the radiochemistry involved with radionuclide production to bolster current production and separation techniques and produce these radionuclides on a large scale. From a theranostics standpoint, identification is needed of chemically and dosimetrically compatible radioisotopes that can be labeled for both imaging and therapy.

The future of TRT in HNSCC relies on identification of promising targets and biomarkers followed by preclinical and clinical trials, incorporating a theranostic approach with a comparison with current imaging and treatment techniques that are already in place. Advancement relies on identifying treatments that are advantageous over the current standard of care and that concurrently increase the patient’s quality of life. Success of TRT in HNSCC relies on a balance between developing a stronger knowledgebase of the predictive biomarkers and their downstream signaling pathways to better predict successful target-target vector interactions, as well as identification of radiometals that will provide the necessary absorbed dose to successfully damage the tumor cells while minimizing the dose to healthy tissue. Numerous mAbs and small molecules have been discussed in this review along with their binding affinities to the EGFR family. By leveraging the EGFR expressions in the tumor, the aforementioned target vectors with high binding affinities could be radiolabeled with the potential for success in TRT.

## 7 Conclusion

HNSCC is the most common type of cancer arising in the head and neck and can develop in the oral cavity, pharynx, nasal cavity, paranasal sinuses, salivary glands, and larynx (7, 8). The current standard of care is either surgical resection followed by adjuvant radiation and chemotherapy or definitive chemoradiation and often comes with considerable limitations and toxicities. The significant burden of morbidity and mortality associated with HNSCC highlights the urgency to explore innovative therapeutic strategies that can enhance treatment efficacy and improve the patient’s quality of life.

The discovery of immunotherapy and the role of the immune system in the treatment of HNSCC has been the focus of early efforts in treating this disease. ICIs have shown promise in the treatment of HNSCC since the 2016 FDA approvals of pembrolizumab and nivolumab for patients with recurrent or metastatic disease, followed by the 2019 approval of pembrolizumab as a first-line therapy. In addition to ICIs such as PD-1 and PD-L1, molecular targets in the EGFR family (EGFR, HER2, and HER3) could be considered a potential focal point for targeted therapy. Preclinical and clinical studies including the EGFR-targeting mAbs cetuximab and panitumumab have demonstrated clinical efficacy (42, 51, 89, 90). Although studies are limited to date, the molecular targets discussed in this review show promise towards HNSCC treatment via TRT. Through the development of radiopharmaceuticals that are able to specifically target molecular markers that are overexpressed on cancer cells, ionizing radiation can be delivered directly to cancer cells while sparing healthy tissue. This method addresses one of the major challenges of traditional therapies, and this potential treatment alternative could substantially reduce the cytotoxicity associated with standard chemotherapy, offering patients a treatment option with fewer adverse effects and improved overall well-being. Current immunoPET advancements in HNSCC research have leveraged the nuclear properties of radionuclides such as ^89^Zr and ^64^Cu conjugated to EGFR-targeting mAbs to obtain high-resolution images of tracer uptake in HNSCC patients. Thus far, some labeled mAbs have proven successful in identifying primary or metastatic lesions and have been deemed clinically safe. These mAbs could be improved upon by developing radiolabeled versions for diagnostic imaging and TRT. Few radioimmunotherapy studies have been conducted for HNSCC *in vivo*, and no FDA-approved radiolabeled antibodies exist for the treatment of HNSCC, although some are approved for use in other types of disease. This existing use case indicates a potential avenue of novel scientific developments with the potential to significantly improve patient care.

The information offered in this review highlights the promising molecular targets within the EGFR family, including EGFR, HER2, and HER3, as well as ICIs such as PD-1 and PD-L1, which represent potential molecular targets for TRT. Preclinical and clinical evidence shows the growing body of research that supports the feasibility and potential benefits of this therapeutic approach, although much still needs to be learned at the molecular level. In combination with traditional treatment modalities, the application of new inhibitors, mAbs, and small molecules in targeting holds great promise and can give insight into more knowledge of the disease. Identification of predictive effectors is of high demand to identify patients who may be candidates for these novel treatments and who may see more favorable treatment outcomes than the current standard of care.

Although progress has been made in developing targeted therapies for HNSCC, this review underscores the need for extensive and rigorous research in the field of TRT. The exploration of predictive biomarkers, a deep understanding of treatment mechanisms, optimization of treatment protocols, and the development of radiopharmaceuticals specifically tailored for HNSCC are all areas that require focused attention. The integration of TRT into the treatment landscape of HNSCC offers hope for patients and clinicians alike. The ongoing commitment to research, innovation, and collaboration between multidisciplinary teams holds the promise of transforming HNSCC treatment, ultimately leading to improved outcomes, prolonged survival, and enhanced quality of life for individuals battling this challenging disease. As the field continues to evolve, TRT stands as a beacon of progress in the pursuit of more effective and less toxic treatment options for HNSCC patients.
